# Risk of Revision Surgery and Manipulation Under Anesthesia in Patients With Cannabis Use Disorder Undergoing Total Knee Arthroplasty

**DOI:** 10.7759/cureus.21016

**Published:** 2022-01-07

**Authors:** Senthil Sambandam, Varatharaj Mounasamy, Sudhakar Selvaraj, Dane Wukich

**Affiliations:** 1 Orthopedics, University of Texas Southwestern Medical Center, Dallas, USA; 2 Psychiatry, University of Texas Health Science Center at Houston McGovern Medical School, Houston, USA

**Keywords:** substance use disorder (sud), revision, manipulation, cannabis, total knee arthroplasty

## Abstract

Background: Total knee arthroplasty (TKA) is a very common orthopedic procedure and with legalization of cannabis in many states, orthopedic surgeons are frequently encountering patients undergoing TKA with preoperative cannabis use disorder. There is conflicting and limited evidence on the impact of preoperative cannabis use and postoperative outcome.

Methods and materials: In this study we retrospectively reviewed PearlDiver insurance database and analyzed the characteristics of the cannabis use in TKA patients and the impact of preoperative diagnosis of cannabis use disorder on postoperative risk of manipulation under anesthesia (MUA) within one year and revision risk within two years. We compared our results to a matched sample of opioid use patients and standard TKA patients.

Result: Our study shows that cannabis use disorder was found in less than 1% of the patients undergoing total knee replacement. We identified 278 patients with cannabis use disorder undergoing TKA. More than 90% of the patients are between 40 to 69 years. There was no statistically significant gender difference noted with 130 males and 148 females. Matching sample analysis showed that the risk of MUA and risk of revision in these cannabis use patients are comparable to standard knee replacement patients and in TKA patients taking preoperative opioids.

Conclusion: Retrospective database review failed to identify any increased risk of MUA or two-year revision in cannabis use TKA patients.

## Introduction

More than 30 states have legalized the medical use of marijuana and several states have legalized the recreational use of cannabis [1]. A recent survey of 43 million people in United States showed that nearly 16% have reported using some form of cannabis at least once in the last year. Another 1.6% has reported cannabis dependence disorder [2,3]. Orthopedic surgeons are increasingly seeing patients in the clinic with self-reported medical or recreational use of cannabis, positive cannabis in the urine during preoperative drug screening or with history of cannabis dependence [4,5]. There is extensive data on the psychotropic and pharmacological effect of cannabis and moderate data on the medical and psychiatric complications in patients on cannabis. However, there is extremely limited data on the impact of cannabis use in surgical patients, especially those undergoing elective orthopedic procedures. Total knee arthroplasty (TKA) is one of the most commonly performed orthopedic procedures, and there is conflicting and limited data about cannabis use in TKA patients. Some studies showed increased postoperative complications following cannabis use while others show no risk associated with preoperative cannabis use [6-8]. The aim of this study was to evaluate the risk of revision and manipulation under anesthesia following TKA in patients with preoperative cannabis use disorder compared to patients with preoperative opioid use in a large database population.

## Materials and methods

In this retrospective study, we used the PearlDiver patient record database (www.pearldiverinc.com, Colorado Springs, CO, USA). PearlDiver is a commercially available database and contains data from Medicare and several different private insurers. The data in PearlDiver has been extensively used in orthopedic research. The database is compliant with the Health Insurance Portability and Affordability Act (HIPPA) and has more than 30 million patient records. We used the MSOrtho30 dataset that included data from Medicare, Medicaid and several private insurers and included all patients from January 2010 until December 2018. Patients undergoing TKA were identified using CPT code 27447. We identified all patients who had a documented diagnosis of cannabis use (ICD9-304.2 and ICD9-305.3) within two years before TKA from the population of TKA. We also identified all patients taking opioids at some point within two years prior to TKA. Our primary outcome measure was to assess the risk of manipulation due to stiffness at three months, six months and 12 months. A secondary outcome measure was to compare the revision rate in cannabis TKA patients, opioid TKA patients and TKA patients who used neither cannabis nor opioids prior to surgery. 

For the purposes of this study, we defined opioid use as the use of opioids anytime for the two years prior to TKA. This group was then matched to the TKA with cannabis use population with respect to age, gender, Charleston Comorbidity Index (CCI), Elixhauser comorbidity index (ECI), and smoking. This matching yielded a total of 276 patients in each group. We then compared the difference in the manipulation rate and two-year revision rate between these two groups.

Statistical methods

Statistical analysis was performed using R statistical program through PearlDIver and Statistical Package for Social Sciences (SPSS; IBM Corp., Armonk, NY, USA). We used descriptive statistics for analysis of baseline demographic variables including age, gender distribution, prevalence of cannabis use and opioid use in the population and postoperative incidence of manipulation under anesthesia (MUA) and revision. Alpha level at 0.05 was considered significant for both numerical and binomial data. Chi square analysis was used to find out the significance of cannabis use and opioid use on the postoperative risk of MUA within one year and revision within two years. Matching was done using the random matching option in the PearlDiver software. Odds ratio (OR) was calculated. All tests were conducted with a significance level of 0.05. Associations between independent variables and dichotomous outcome variables were assessed using OR and corresponding 95% confidence intervals (CI) were determined.

## Results

Our database search with CPT code 27447 identified 232,014 patients who underwent total knee arthroplasty for the time period from January 2010 to December 2018 (Table 1). Two hundred seventy-eight patients (0.1%) were documented to be taking cannabis within two years before their total knee arthroplasty, of which there were 130 males and 148 females. Almost 92% of the TKA patients using cannabis were between the ages of 45 and 69 years, and more than 50% of users were between 50 and 59 years old. We identified 133,709 patients (57.6%) with documented opioid use within two years before total knee arthroplasty.

**Table 1:** MUA and revision in an unmatched cohort of all patients undergoing total knee arthroplasty. MUA: manipulation under anesthesia

Patient Characteristics	Total knee arthroplasty for all patients	MUA at 90 days	MUA rate	MUA at 180 days	MUA rate	MUA at 1 year	MUA rate	Revision at 2 years	Revision rate
Total	232014	6990	3.01%	8774	3.78%	9156	3.94%	5361	2.31%
Female	147734	4584	3.10%	5857	3.96%	6125	4.14%	3157	2.13%
Male	84279	2406	2.85%	2917	3.46%	3031	3.59%	2204	2.61%
35-39	628	52	8.28%	61	9.71%	66	10.50%	39	6.21%
40-44	2171	192	8.84%	221	10.17%	230	10.59%	136	6.26%
45-49	6727	453	6.73%	544	8.08%	572	8.50%	293	4.35%
50-54	17174	983	5.72%	1221	7.10%	1280	7.45%	644	3.74%
55-59	30666	1366	4.45%	1709	5.57%	1785	5.82%	907	2.95%
60-64	42664	1373	3.21%	1735	4.06%	1808	4.23%	998	2.33%
65-69	45155	1144	2.53%	1454	3.22%	1517	3.35%	938	2.077%
70-74	59080	1004	1.69%	1278	2.16%	1335	2.25%	960	1.62%
75-79	33359	402	1.20%	532	1.59%	558	1.67%	468	1.40%
80-84	3473	24	0.6%	30	0.86%	30	0.86%	18	0.51%

MUA after TKA in cannabis patients

Out of the total 231 375 patients with no documented use of cannabis, 9143 patients (3.9%) underwent MUA at the end of one year, 6107 of these 9143 (66.8%) patients were females. Out of 278 patients with a diagnostic code of cannabis use, less than 11 patients underwent MUA within 90 days (exact number not given due to privacy reasons), 13 patients (4.6%) underwent MUA in 180 days and 13 patients (4.6%) underwent MUA in 365 days. MUA rate was highest in females at 180 days and 365 days (11 out of 13) (Table 2).

**Table 2:** MUA and revision in an unmatched cohort of all patients undergoing total knee arthroplasty and having cannabis use disorder. NA: not available, MUA: manipulation under anesthesia -1: Pearldiver reports as -1 if the number is less than 11

Patient Demographics	Total knee arthroplasty in Cannabis group	MUA at 90 days	MUA rate at 90 days	MUA at 180 days	MUA rate at 180 days	MUA at 1 year	MUA rate at 1 year	Revision at 2 years	Revision rate at 2 years
Total	278	-1	NA	13	4.67%	13	4.67%	14	5.0%
Female	148	-1	NA	11	7.43%	11	7.43%	-1	NA
Male	130	-1	NA	2	1.53%	2	1.53%	-1	NA
35-39	-1	-1	NA	-1	NA	-1	NA	-1	NA
40-44	-1	-1	NA	-1	NA	-1	NA	-1	NA
45-49	38	-1	NA	-1	NA	-1	NA	-1	NA
50-54	79	-1	NA	-1	NA	-1	NA	-1	NA
55-59	76	-1	NA	-1	NA	-1	NA	-1	NA
60-64	47	-1	NA	-1	NA	-1	NA	-1	NA
65-69	14	-1	NA	-1	NA	-1	NA	-1	NA
70-74	-1	-1	NA	-1	NA	-1	NA	-1	NA
75-79	-1	-1	NA	-1	NA	-1	NA	-1	NA
80-84	-1	-1	NA	-1	NA	-1	NA	-1	NA

MUA after TKA in opioid patients

We identified 133,709 patients with documented opioid use within two years before total knee arthroplasty. The MUA rate was 3.06% (4096 patients) at 90 days, 3.86% (5168 patients) at 180 days and 4.04% (5407 patients) at 365 days (Table 3).

**Table 3:** MUA and revision in an unmatched cohort of patients undergoing total knee arthroplasty with preoperative opioid use. MUA: manipulation under anesthesia

Patient Demographics	Total knee arthroplasty in opioid group	MUA at 90 days	MUA rate	MUA at 180 days	MUA rate at 180 days	MUA at 1 year	MUA rate at 1 year	Revision at 2 years	Revision rate at 2 years
Total	133709	4096	3.06%	5168	3.86%	5407	4.04%	3503	4.04%
Female	87943	2768	3.14%	3565	4.05%	3732	4.24%	2135	4.24%
Male	45766	1328	2.90%	1603	3.50%	1675	3.65%	1368	3.65%
35-39	478	38	7.94%	46	9.62%	50	10.46%	31	10.46%
40-44	1569	137	8.73%	157	10.00%	165	10.51%	105	10.51%
45-49	4702	305	6.48%	365	7.76%	391	8.31%	235	8.31%
50-54	11419	642	5.62%	798	6.98%	836	7.32%	471	7.32%
55-59	19152	830	4.33%	1052	5.49%	1098	5.73%	620	5.73%
60-64	25389	799	3.14%	1020	4.017%	1067	4.20%	655	4.2%
65-69	25580	611	2.38%	788	3.08%	822	3.21%	567	3.21%
70-74	28885	483	1.67%	615	2.12%	645	2.23%	542	2.23%
75-79	18570	231	1.24%	306	1.64%	319	1.71%	289	1.71%
80-84	1748	14	0.80%	18	1.02%	18	1.02%	12	1.02%

Two-year revision rate after TKA in cannabis patients, opioid patients and non-cannabis patients

Out of the 278 patients diagnosed with cannabis use, 14 patients underwent revision (5.0%). The revision rate in the opioid group was 2.8% (3503 patients). The revision rate in the total knee patient without documented use of cannabis was only 2.3% (5347 patients). The exact age group and sex distribution were unavailable since each group's number was less than 11.

Matched samples analysis of MUA and revision in the cannabis group compared to the non-cannabis group

We matched the 278 patients who used cannabis before TKA to the population of patients who underwent TKA but had no documented cannabis use in the two years before surgery; patients were matched for age, sex, smoking status, CCI, and ECI. The match yielded 262 patients in each group, with 141 females (53.8%) and 121 males (46.2%) and 208 smokers (79.3%) and 54 non-smokers (20.7%).

Twelve patients (5%) underwent MUA at one year in the matched cannabis group (Table 4) and 17 patients (6.4%) underwent MUA in the matched non-cannabis group and this difference was not statistically significant (P=0.34). Fourteen patients (5.3%) underwent revision in the matched non-cannabis group and 15 patients (5.7%) underwent revision in the cannabis group. This difference was not statistically significant (P=0.84).

**Table 4:** MUA and revision risk in matched cohort of cannabis and standard TKA. MUA: manipulation under anesthesia, TKA: total knee arthroplasty

Revision and MUA risk	Cannabis group	No cannabis no opioid group	p-value
MUA in 1 year	12	17	OR- 0.69 (0.32 to 1.47) p =0.34
Revision in two years	15	14	OR- 1.07 (0.50 to 2.27) P =0.848

Matched samples analysis of MUA and revision in the cannabis group compared to the opioid group

We matched the 278 patients who used cannabis before TKA to the population of patients who underwent TKA but had documented use of opioids within two years before surgery; patients were matched for again age, sex, CCI and ECI (Table 5). The match yielded 276 patients in each group, with 148 females (53.6%) and 128 males (46.4%).

**Table 5:** Characteristics of the matched cohort of cannabis and opioid use TKA. TKA: total knee arthroplasty -1: Pearl driver reports it as -1 if the number is less than 11.

Patient Demographics	Matched samples characteristics
Total	276
Female	148
Male	128
35-39	-1
40-44	-1
45-49	38
50-54	79
55-59	76
60-64	47
65-69	14
70-74	-1
75-79	-1
ECI	00: 64 01: 74 02: 49 03: 27 04: 18 05: 14 06: -1 07: 11 08: -1 09: -1 11: -1 12: -1 21: -1
CCI	01: -1 02: -1 03: -1 04: -1 05: 17 06: 16 07: 31 08: 27 09: 27 10: 31 11: 19 12: 27 13: 20 14: 11 15: -1 16: -1 17: -1 18: -1 19: -1 20: -1 21: -1 23: -1

Twelve patients (4.3%) underwent MUA in the cannabis group (Table 6) and 13 patients (4.7%) underwent MUA in the opioid group and this difference was not statistically significant (p=0.83). Eleven patients (3.9%) underwent revision at two years in the opioid group and 14 patients (5%) underwent revision in the cannabis group. This difference was also not statistically significant (p=0.539).

**Table 6:** MUA and revision risk in a matched cohort of cannabis and opioid users. MUA: manipulation under anesthesia

MUA and revision risk	Cannabis group	Opioid group	p-value
MUA in 1 year	12	13	OR- 0.91 (0.41- 2.05) p =0.837
Revision in two years	14	11	OR- (0.57- 2.88) p =0.539

## Discussion

Our study showed that cannabis use was reported in only 1.1% of the patients undergoing total knee replacement. Similar low prevalence was reported by other authors using large databases. A study based on national inpatient samples showed a prevalence of 0.04% [3]. A study based on National Hospital discharge survey by Best et al. [4] database showed a 0.01% prevalence of cannabis use disorder. A previous PearlDiver database study by Law et al. [6] revealed a prevalence of 0.7% which is similar to our study prevalence. A single-institution study by Denduluri et al. [8] showed preoperative cannabis use prevalence of around 15%. Similar high perioperative use of cannabis was reported based on single-institution data by Runner et al. [5]. These higher numbers from single-institution studies were either due to the inclusion of self-reported cannabis use and routine preoperative screening in single-institution samples or due to lack of laboratory screening data in the database samples. Also, the incidence of cannabis dependence seen in our TKA patient group was comparable to the population prevalence of 1.6% reported in the past [2].

In our study, there was no significant gender difference in prevalence of cannabis use disorder in TKA patients and most patients with documented cannabis use disorder before TKA were between 45 and 69 years old. This age group is similar in the studies reported by Goel et al. [1] and Best et al. [4]. The average age of the patient in the cannabis use population reported by Goel et al. was 37 years and by Best et al. was 51.6 years. This explains the lower prevalence of preoperative comorbidities in cannabis and another drug use group. The difference in the age distribution and prevalence of comorbidities highlights the importance of matching as shown by Goel et al. [1]. Before matching the cannabis use, cannabis group was noted to have significantly higher odds of developing several complications including myocardial infarction (MI), cerebral vascular accident (CVA) and respiratory failure, however after matching only the odds of developing MI was significant.

Several short term complications like infection, deep vein thrombosis (DVT), pulmonary embolism (PE), readmission rate, cost of care and discharge against medical advice were analyzed by various authors and noted to be higher in the cannabis group compared to the non-cannabis group [6-9]. However, risk of MUA has not been reported by any of the previous studies. In our study, risk of manipulation in the standard total knee population at one year was 3.94%. The risk of manipulation in the cannabis group was 4.64% with an increased odd of 1.19 times for MUA. However, when we matched the two populations and eliminated the variables known to increase the risk of MUA, the odds of MUA were comparable in the cannabis group and standard knee group. This supports the finding of Jennings et al. [7] who found no difference in the postoperative range of movements in cannabis and non-cannabis group.

Revision after TKA in patients using cannabis has been studied by a small number of researchers with conflicting results [6-8]. Law et al. [6] in his database research using PearlDiver reported higher complications and revision after TKA in patients with cannabis use disorder. In contrast, Jennings et al. in their single institution-based study noted no increase in the reoperation rate [7]. Similarly, Denduluri et al. in another single-institution study noted no increase in the reoperation rate, infection or Veterans Affairs Surgical Quality Improvement Program (VASQIP) capture complications [8]. In our study the unmatched two-year revision risk is significantly higher in the cannabis group compared with standard group with an OR of 2.24. However, when we matched for all comorbidities known to increase the risk of revision, the risk of revision in the cannabis group was not significantly different compared to standard group much like the findings of Jennings et al. [7] and Denduluri et al. [8].

## Conclusions

Our study fails to show any increased risk of MUA within one year in cannabis users and no increased risk of revision within two years. Even though cannabis use among patients continues to increase in the general population, this large database study did not identify an increased risk for MUA or revision surgery after TKA. This information should be taken into consideration while implementing preoperative routine screening protocols in health care to avoid unnecessary hospital resource utilization without any added benefits.
